# Musical components important for the Mozart K448 effect in epilepsy

**DOI:** 10.1038/s41598-021-95922-7

**Published:** 2021-09-16

**Authors:** Robert J. Quon, Michael A. Casey, Edward J. Camp, Stephen Meisenhelter, Sarah A. Steimel, Yinchen Song, Markus E. Testorf, Grace A. Leslie, Krzysztof A. Bujarski, Alan B. Ettinger, Barbara C. Jobst

**Affiliations:** 1 Department of Neurology, Geisel School of Medicine at Dartmouth, Hanover, NH USA; 2 Department of Music at Dartmouth College, Hanover, NH USA; 3 Department of Computer Science at Dartmouth College, Hanover, NH USA; 4 Department of Neurology, Dartmouth–Hitchcock Medical Center, 1 Medical Center Drive, Lebanon, NH 03766 USA; 5 Thayer School of Engineering at Dartmouth College, Hanover, NH USA; 6 Department of Music, Georgia Institute of Technology, Atlanta, GA USA; 7 United Diagnostics, New Hyde Park, NY USA

**Keywords:** Epilepsy, Neurology, Computational neuroscience

## Abstract

There is growing evidence for the efficacy of music, specifically Mozart’s Sonata for Two Pianos in D Major (K448), at reducing ictal and interictal epileptiform activity. Nonetheless, little is known about the mechanism underlying this beneficial “Mozart K448 effect” for persons with epilepsy. Here, we measured the influence that K448 had on intracranial interictal epileptiform discharges (IEDs) in sixteen subjects undergoing intracranial monitoring for refractory focal epilepsy. We found reduced IEDs during the original version of K448 after at least 30-s of exposure. Nonsignificant IED rate reductions were witnessed in all brain regions apart from the bilateral frontal cortices, where we observed increased frontal theta power during transitions from prolonged musical segments. All other presented musical stimuli were associated with nonsignificant IED alterations. These results suggest that the “Mozart K448 effect” is dependent on the duration of exposure and may preferentially modulate activity in frontal emotional networks, providing insight into the mechanism underlying this response. Our findings encourage the continued evaluation of Mozart’s K448 as a noninvasive, non-pharmacological intervention for refractory epilepsy.

## Introduction

Epilepsy impacts approximately 1% of the global population, and of these people, 1/3 suffer from medication-resistant or refractory epilepsy^1^. Besides seizures and their associated comorbidities, persons with epilepsy experience interictal epileptiform discharges (IEDs). IEDs arise from the brief, synchronous firing of neural populations that are typically involved with epileptic networks^2^. These IEDs are known epileptic biomarkers that are associated with seizure frequency and impaired cognition^3–6^. Thus, IED-related interventions may provide insight into novel therapies for epilepsy and its related comorbidities.

An IED-related intervention with accumulating evidence is the use of music as a noninvasive, non-pharmacologic form of neuromodulation^7,8^. Specifically, Mozart’s Sonata for Two Pianos in D Major (K448) has been shown to reduce ictal and interictal epileptiform activity in several scalp-EEG and fMRI studies^9–12^. While effect sizes varied, a meta-analysis demonstrated that approximately 84% of subjects had significant IED reductions during Mozart’s K448^13^. This reputed “Mozart K448 effect” was first described in 1993 by Rauscher et al.^14^ when they demonstrated enhancement on a spatial task during exposure to K448. Later, Hughes et al. (1998)^15^ were the first to witness the “Mozart K448 effect” in persons with epilepsy by showing that K448 was associated with reduced epileptiform activity. Following Hughes et al.’s discovery, there has been continued support for the “Mozart K448 effect” in epilepsy research—generally demonstrating that exposure to K448 was associated with some therapeutic reduction in seizures and IEDs^7,9,12,16,17^.

Apart from one other composition—Mozart’s Piano Sonata in C Major (K545)—the therapeutic properties of K448 could not be replicated with other musical stimuli^18^. Stimuli previously tested were other Mozart compositions^16^, Beethoven’s Fur Elise^19^, and a string version of K448^10^. This led to several theories about the mechanisms underlying Mozart’s therapeutic effects for epilepsy; however, the specific properties driving the “Mozart K448 effect” remain unknown. Consequently, there is a general reluctance to fully accept this effect due to the unknown mechanism of K448 and to heterogeneous past findings that are likely linked with the use of different study protocols and inferior imaging modalities. The latter limitation is noteworthy, as scalp-EEG is much less sensitive for quantifying epilepsy-related outcomes, especially interictal events^20,21^.

Our previous work demonstrated that 40 Hz auditory stimulation could reduce IEDs in subjects with refractory epilepsy and high baseline IED rates^22^. Historically, the relevance of gamma sensory stimulation emerged from findings of reduced gamma oscillations in humans with Alzheimer’s disease (AD)^23^. This was followed by observations of improved disease states (e.g., AD^24–26^ and stroke^27^) after exposure to exogenous gamma stimulation. Lin et al. (2010)^10^ even demonstrated that musical stimuli with more fundamental tones (i.e., higher gamma power) reduced the number of epileptiform discharges. A major pitfall to this noninvasive intervention is that while the 40 Hz tone could effectively reduce IEDs in refractory epilepsy, it was not especially pleasant to listen to for a prolonged time.

In this study, we evaluate the use of Mozart’s K448 to see (1) if we can validate previous scalp-EEG findings with intracranial Stereo-EEG in adults with refractory epilepsy, (2) if there is a temporal dependence for eliciting the “Mozart K448 effect”, and (3) if the “Mozart K448 effect” is associated with preferential brain networks. We also examined if preferred music and music with enhanced gamma frequencies (i.e., either gamma-matched to K448 or gamma-boosted) could elicit a therapeutic response; this was motivated by our past findings and the theory that increased fundamental frequencies may be beneficial for epilepsy^10,22^. We hypothesized that eliciting the “Mozart K448 effect” would be dependent on a longer stimulus duration and prolonged internal musical segments. This is based on the theory that emotional responses result from positive reward prediction errors^28^. Further, we expected this effect would extend to regions outside of the primary auditory pathways, owing to past observations of music and its involvement with higher order systems (e.g., emotion and mirror neurons)^29^. This research may guide future work in uncovering how Mozart’s K448 elicits therapeutic responses, which may facilitate the development of novel, noninvasive music therapies for refractory epilepsy.

## Results

### Detecting interictal activity in subjects with refractory epilepsy

**Figure 1:**
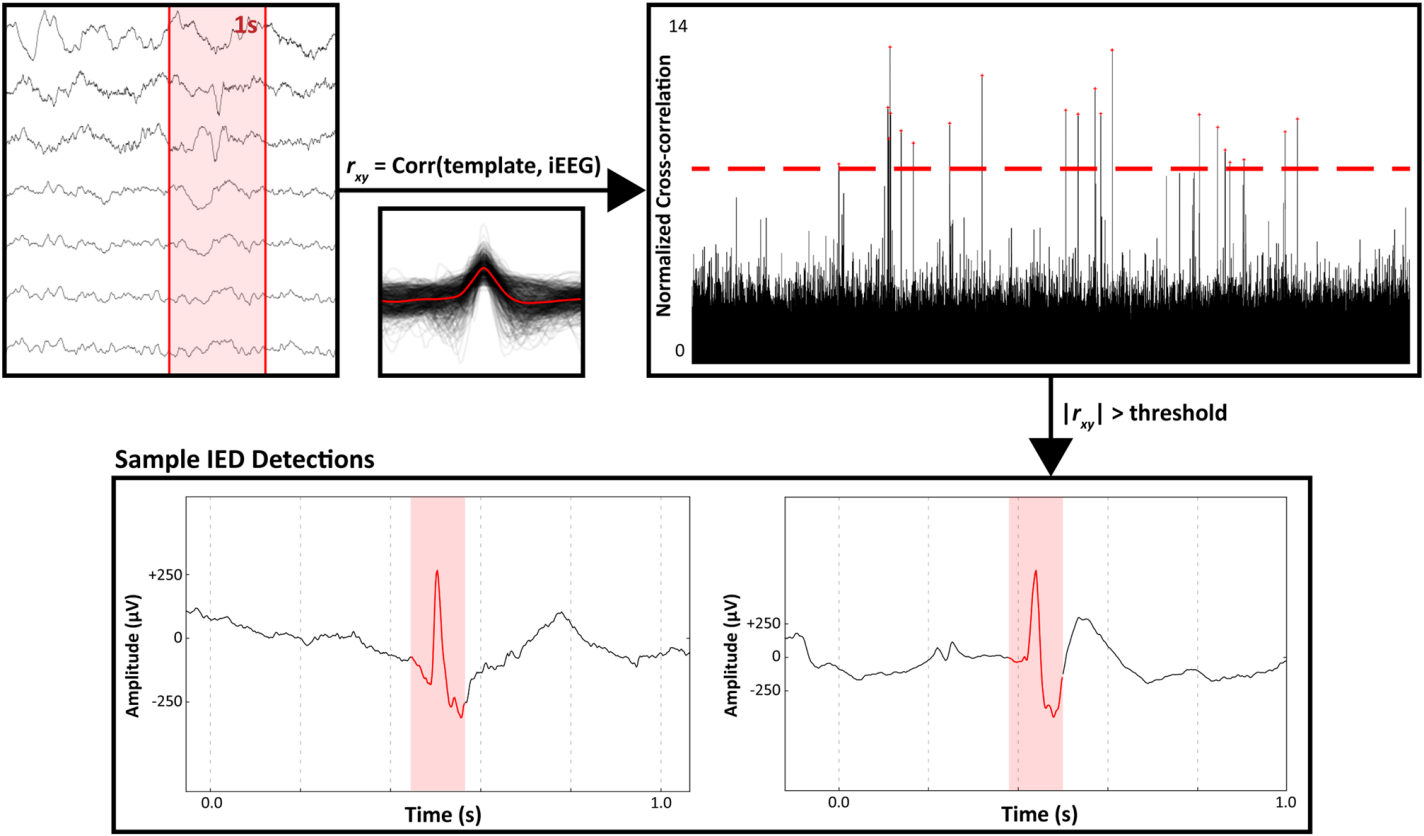
Automated spike detector pipeline. A template-matching IED detector first cross-correlated a 60-ms triangular template with preprocessed Stereo-EEG, then normalized the cross-correlation by the median standard deviation from 1-s sliding windows. The absolute value of the normalized cross-correlation was then used to mark local peaks above a specified threshold as IEDs.

**Figure 2:**
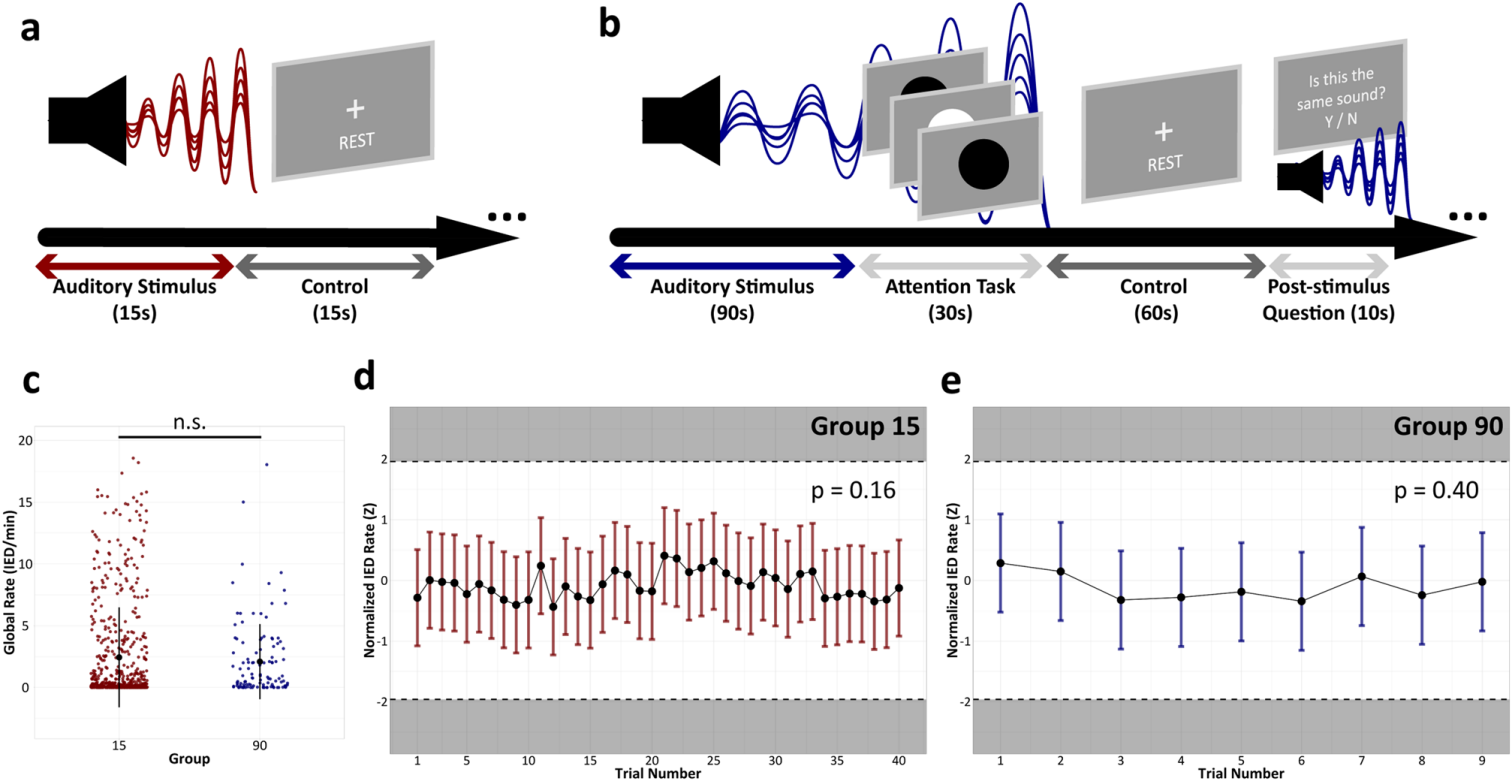
Task structure and validation method. (**a**) Trials consisted of auditory stimuli selected randomly without replacement, each presented for 15-s, followed by a 15-s rest period (“Group 15”). (**b**) Trials consisted of musical stimuli selected randomly without replacement, where a simultaneous attention task was performed during the final 30-s of each auditory stimulus. This was followed by a 60-s control period and a True/False question assessing whether the subject attended to the auditory stimulus (“Group 90”). (**c**) A marginal model (GEE) revealed a nonsignificant difference in global normalized IED rates between the control periods of each study group (*p* = 0.92). RM-ANOVA on z-scored IED rates demonstrated no significant fluctuation in IEDs between control periods for Group 15 (*p* = 0.16) (**d**) and Group 90 (*p* = 0.40) (**e**); means and standard deviations are depicted.

An automated template-matching interictal epileptiform discharge detector was utilized to calculate subject-specific IED rates (Fig. 1). We recruited 16 neurosurgical subjects undergoing clinical monitoring for refractory epilepsy to participate in sessions of a music task (Fig. 2a,b). To determine if the duration of exposure was an important factor for eliciting the “Mozart K448 effect”, stimuli were presented for either 15-s (“Group 15”) or 90-s (“Group 90”). Subjects in Group 15 had a mean age of 43.75 (SD 16.46), an average normalized baseline IED rate of 1.23 (SD 1.09), and 50% were male. Group 15 subjects had 32.5 (SD 14.40) electrodes implanted in the left hemisphere and 35.86 (SD 12.78) electrodes in the right hemisphere. Subjects in Group 90 had a mean age of 34.88 (SD 10.02), an average normalized baseline IED rate of 1.43 (SD 0.94), and 75% were male. Group 90 subjects had 38 (SD 22.67) electrodes implanted in the left hemisphere and 33.38 (SD 22.77) electrodes in the right hemisphere. Subjects from both groups performed 1.81 (range 1–2, SD 0.40) 25-min sessions on average. Other subject demographic and clinical characteristics are provided in Table 1.

**Table 1:** Subject information.

Subject	SOZ^a^	MRI findings	Handedness	Age	Gender	Normalized rate^b^ (IED/min)	Left channels^c^	Right channels^c^
**Group 15**								
1	Right TC	Right sphenoidal encephalocele, Right temporal encephalocele, Right temporal encephalocele	Left	65	Female	1.81	46	46
2	Right MESIAL	Right mesial temporal sclerosis	Right	39	Male	0.67	32	29
3	Right MESIAL	Right temporal encephalocele, Left mesial temporal sclerosis	Right	38	Female	2.8	38	42
4	Right Inferior FC	Bifrontal gliotic changes	Right	68	Female	0.11	17	47
5	Left HIP	Unremarkable	Left	28	Female	2.65	57	18
6	Right TC	Right Encephalomalacia (temporal, parietal, orbitofrontal)	Right	35	Male	0.49	20	30
7	Right TC, Left Posterior TC	Unremarkable	Right	24	Male	1.26	17	56
8	Right TC, Left TC	Right arachnoid cyst	Right	53	Male	0.034	33	30
**Group 90**								
1	Left TC	Left temporal encephalomalacia, Left hippocampus volume loss	Right	29	Female	2.09	57	0
2	Right TC	Unremarkable	Right	43	Female	0.97	0	61
3	Bilateral MESIAL	Left otomastoid effusion	Right	30	Male	0.48	32	32
4	Left MESIAL	Bilateral inferior frontal and inferior right temporal encephalomalacia	Right	56	Male	1.79	44	44
5	Bilateral MESIAL	Unremarkable	Right	27	Male	3.23	55	53
6	Right MESIAL	Unremarkable	Right	27	Male	1.52	51	45
7	Right MESIAL	Right temporal focal acute parenchymal hemorrhage, Scattered subarachnoid and small volume intraventricular hemorrhages	Right	32	Male	0.88	8	32
8	Left TC	Left posterior hippocampal sclerosis, Left mamillary body atrophy, Left middle cranial fossa arachnoid cyst	Right	35	Male	0.50	57	0

^a^Seizure Onset Zone (SOZ) corresponds to areas that initiated clinical seizures during intracranial monitoring. FC Frontal Cortex, TC Lateral Temporal Cortex, MESIAL Mesial Temporal Cortex.

^b^Time-adjusted baseline IED rates averaged over all contacts with at least one IED during the task window.

^c^Denotes the number of contacts remaining after the exclusion of bad channels and channels outside of co-registered grey matter regions.

### Validation of the control

We first confirmed that Group 15 and Group 90 were comparable by verifying a nonsignificant difference in the global normalized IED rates between the average control periods from each group (*p* = 0.92) (Fig. 2c). Our GEE model indicated that ASM status was a significant confounder (*p* = 0.027); therefore, all future models controlled for ASM status and session time, as these factors were previously shown to influence IED rates^30–32^. Similar GEE models were used to show that there was no significant difference in the global IED rates between the pre-stimulus baseline period and the control period for Group 15 (*p* = 0.82) and Group 90 (*p* = 0.88). Our RM-ANOVA of z-scored IED rates showed no significant fluctuation in IEDs between the control periods of all trials for Group 15 (*p* = 0.16) (Fig. 2d) and Group 90 (*p* = 0.40) (Fig. 2e). Together, these findings supported our use of the nested control periods as a reference in subsequent models.

### Global IED reductions are dependent on the duration of K448

**Figure 3:**
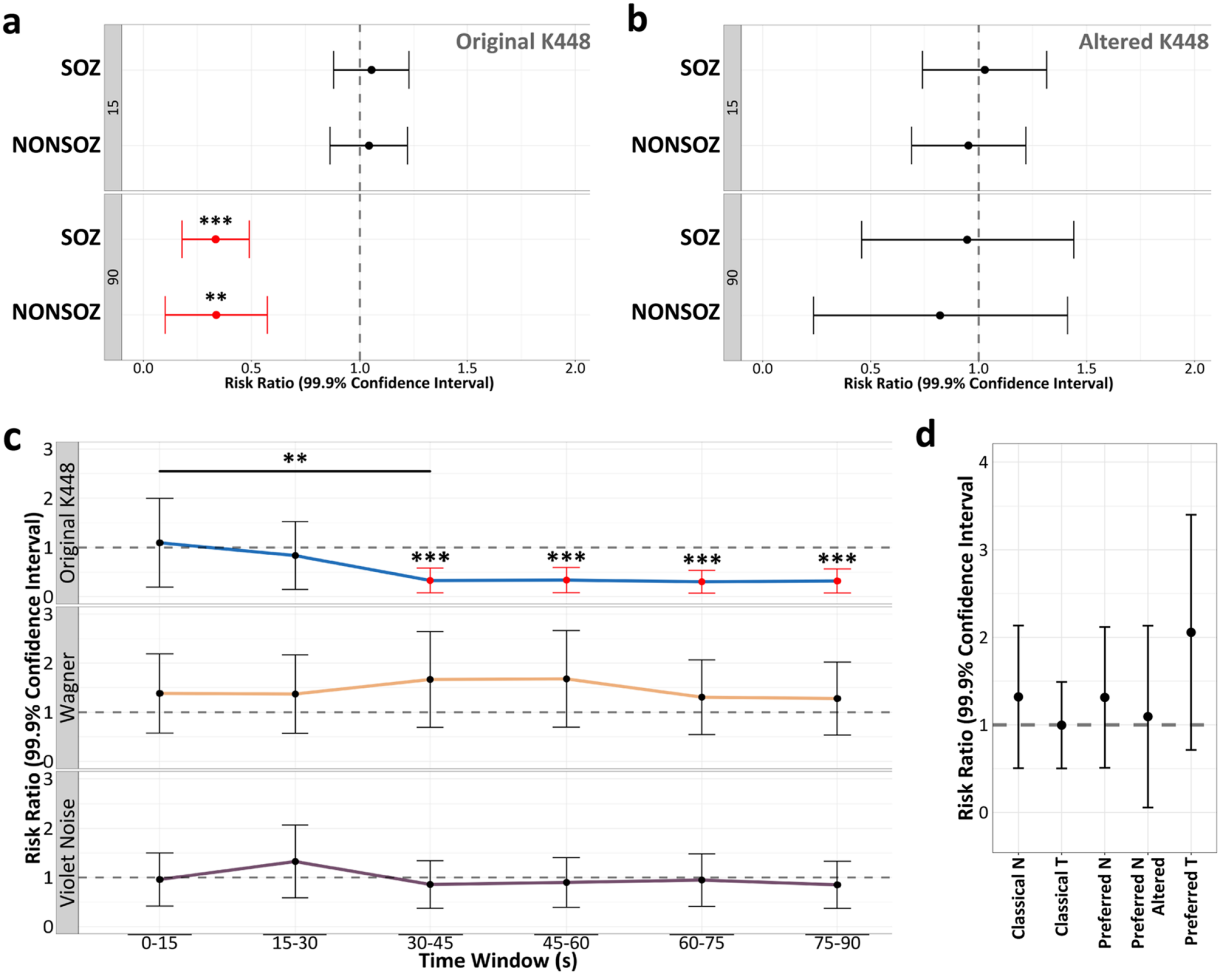
Reduced global IED rates are dependent on the duration of music exposure. (**a**) GEE models showed that the original version of K448 was the only stimulus effective at reducing IEDs with at least 90-s of exposure. (**b**) Nonsignificant reductions were observed for the altered versions of K448 (top = modulatedK448, bottom = filteredK448). (**c**) Partitioning the 90-s window of Mozart’s original K448 revealed that IED reductions only began after 30-s of exposure. There was a significant IED reduction between the 0–15 and 30–45 windows (*p* = 0.004). Control stimuli (musical control = Wagner’s Lohengrin [*Prelude to Act I*], nonmusical control = violet noise) demonstrated nonsignificant IED reductions for each time window. (**d**) All other musical stimuli presented to Group 90 showed nonsignificant IED reductions. “T” or “N” following each song label indicates if the gamma-range auditory modulation spectrum of that song matched (“T”) or did not match (“N”) that of K448. “Altered” indicates signals with secondary gamma modulations. Significance at *p < 0.05, **p < 0.01, ***p < 0.001.

After confirming that the control periods were similar between groups, we could more confidently compare interictal epileptiform responses to auditory stimuli. Our GEE models demonstrated a significant reduction in global IED rates during 90-s of exposure to the original version of K448 both inside and outside of the seizure onset zone (SOZ) (SOZ RR = 0.33, *p* < 0.001; Non-SOZ RR = 0.34, *p* = 0.0013) (Fig. 3a). This effect was only present for the original K448, as we observed a nonsignificant change for the filtered version of K448 with 90-s of exposure (SOZ RR = 0.95, *p* = 0.48; Non-SOZ RR = 0.82, *p* = 0.23) (Fig. 3b). Nonsignificant IED rate reductions were also shown with 15-s of exposure to the original K448 (SOZ RR = 1.05, *p* = 0.65; Non-SOZ RR = 1.04, *p* = 0.76) (Fig. 3a) and the amplitude modulated version of K448 (SOZ RR = 1.03, *p* = 0.93; Non-SOZ RR = 0.96, *p* = 0.55) (Fig. 3b).

In evaluating the data from Group 90, where the 90-s window was divided into six 15-s windows, we revealed that IED rate reductions were only present after at least 30-s of exposure (30–45 s RR = 0.31, *p* < 0.001; 45–60 s RR = 0.34, *p* < 0.001; 60–75 s RR = 0.33, *p* < 0.001; 75–90 s RR = 0.31, *p* < 0.001) (Fig. 3c). Nonsignificant IED reductions were observed for all times less than 30-s (0–15 s RR = 1.09, *p* = 0.99; 15–30 s RR = 0.83, *p* = 0.21) (Fig. 3c). Our paired-sample comparison of the 0–15 window and the 30–45 window corroborated this finding in showing a significant reduction in IEDs (*p* = 0.004) (Fig. 3c).

Applying the same procedure to Wagner’s Lohengrin (*Prelude to Act I*) demonstrated nonsignificant IED reductions for all time windows (0–15 s RR = 1.98, *p* = 0.41; 15–30 s RR = 2.27, *p* = 0.13; 30–45 s RR 2.07, *p* = 0.28; 45–60 s RR = 2.08, *p* = 0.28; 60–75 s RR = 1.84, *p* = 0.73; 75–90 s RR = 1.98, *p* = 0.40) (Fig. 3c). Similarly, violet noise demonstrated nonsignificant IED reductions for all time windows (0–15 s RR = 0.95, *p* = 0.30; 15–30 s RR = 1.18, *p* = 0.98; 30–45 s RR 0.80, *p* = 0.07; 45–60 s RR = 0.82, *p* = 0.08; 60–75 s RR = 0.97, *p* = 0.35; 75–90 s RR = 0.85, *p* = 0.11) (Fig. 3c). Our evaluation of all other musical stimuli presented to Group 90 revealed nonsignificant IED reductions for music from the preferred genre (Preferred T RR = 2.83, *p* = 0.20; Preferred N RR = 1.42, *p* = 0.78; Preferred N Altered RR = 1.51, *p* = 0.15) and the classical genre (Classical T RR = 1.16, *p* = 0.83; Classical N RR = 1.45, *p* = 0.79) (Fig. 3d).

### K448 preferentially reduced IEDs in bilateral frontal regions

**Figure 4:**
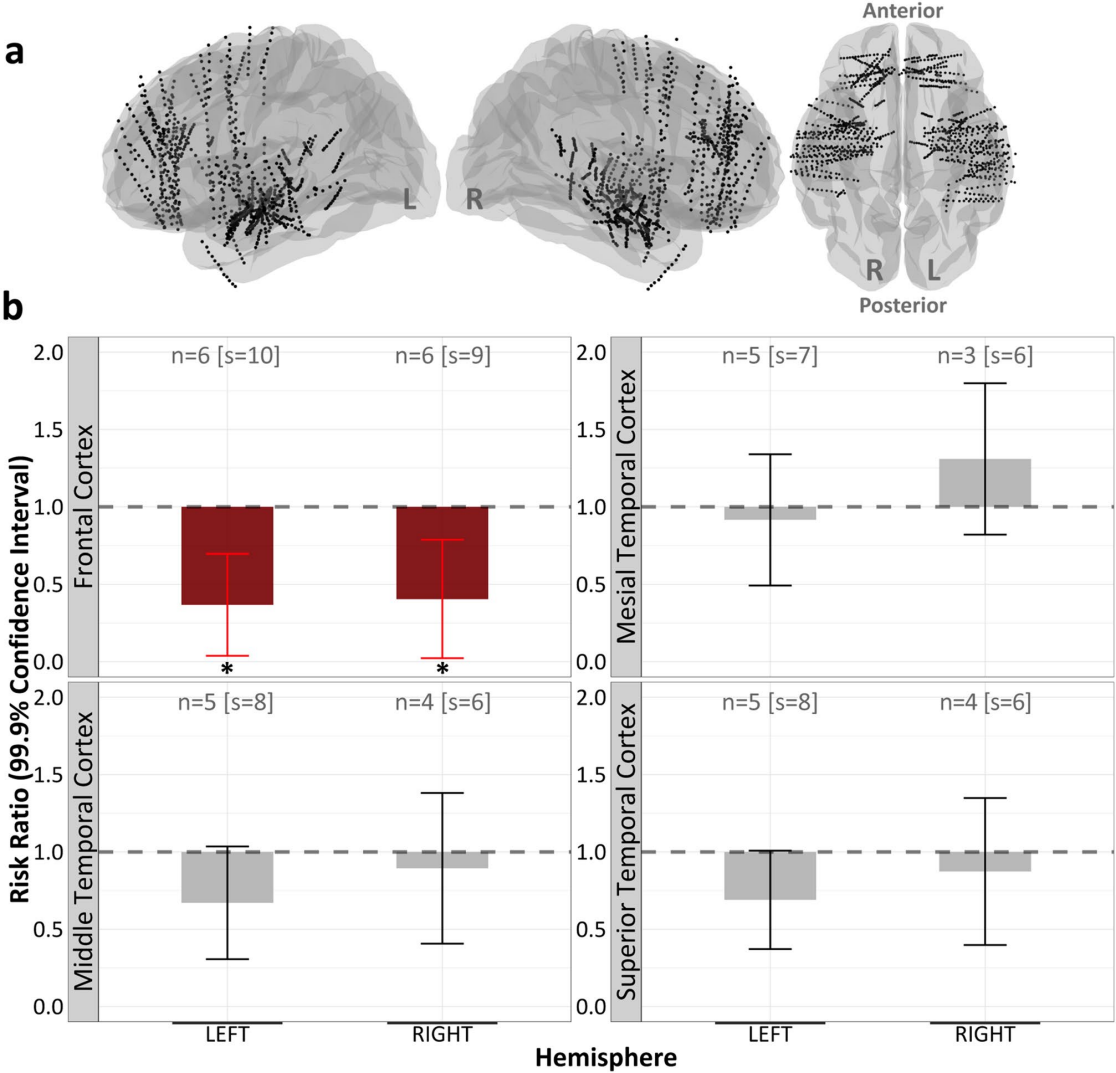
Bilateral frontal regions responded to K448. (**a**) Stereo-EEG electrodes aggregated across subjects. (**b**) Linear mixed models revealed nonsignificant reductions in all regions other than the bilateral frontal cortices (right frontal cortex (FC) % reduction = 59.55, *p* = 0.049; left FC % reduction = 63.25, *p* = 0.017). “n” represents the number of subjects with electrode coverage in the corresponding brain region and “s” represents the number of unique experiment sessions. Significance at *p < 0.05, **p < 0.01, ***p < 0.001.

We next examined region-specific IED rate alterations for regions outside of a subject’s specified SOZ. This was done to see if we could localize the “Mozart K448 effect” in less pathologic brain tissue, identified with implanted intracranial electrodes (Fig. 4a), while also minimizing the impact that subject-specific SOZs had on IED rates. Linear mixed effects models demonstrated significant IED reductions in the bilateral frontal cortices (right frontal cortex (FC) % reduction = 59.55, *p* = 0.049; left FC % reduction = 63.25, *p* = 0.017) (Fig. 4b). Nonsignificant IED reductions were observed for all other brain regions (right superior temporal cortex [STC] % reduction = 12.69, *p* = 0.22; right middle temporal cortex [MTC] % reduction = 10.60, *p* = 0.25; right mesial temporal cortex [Mesial] % reduction = 18.01, *p* = 0.99; left STC % reduction = 31.02, *p* = 0.06; left MTC % reduction = 32.96, *p* = 0.07; left Mesial % reduction = 8.39, *p* = 0.29) (Fig. 4b).

### Transitions from longer K448 segments increased frontal theta activity

**Figure 5:**
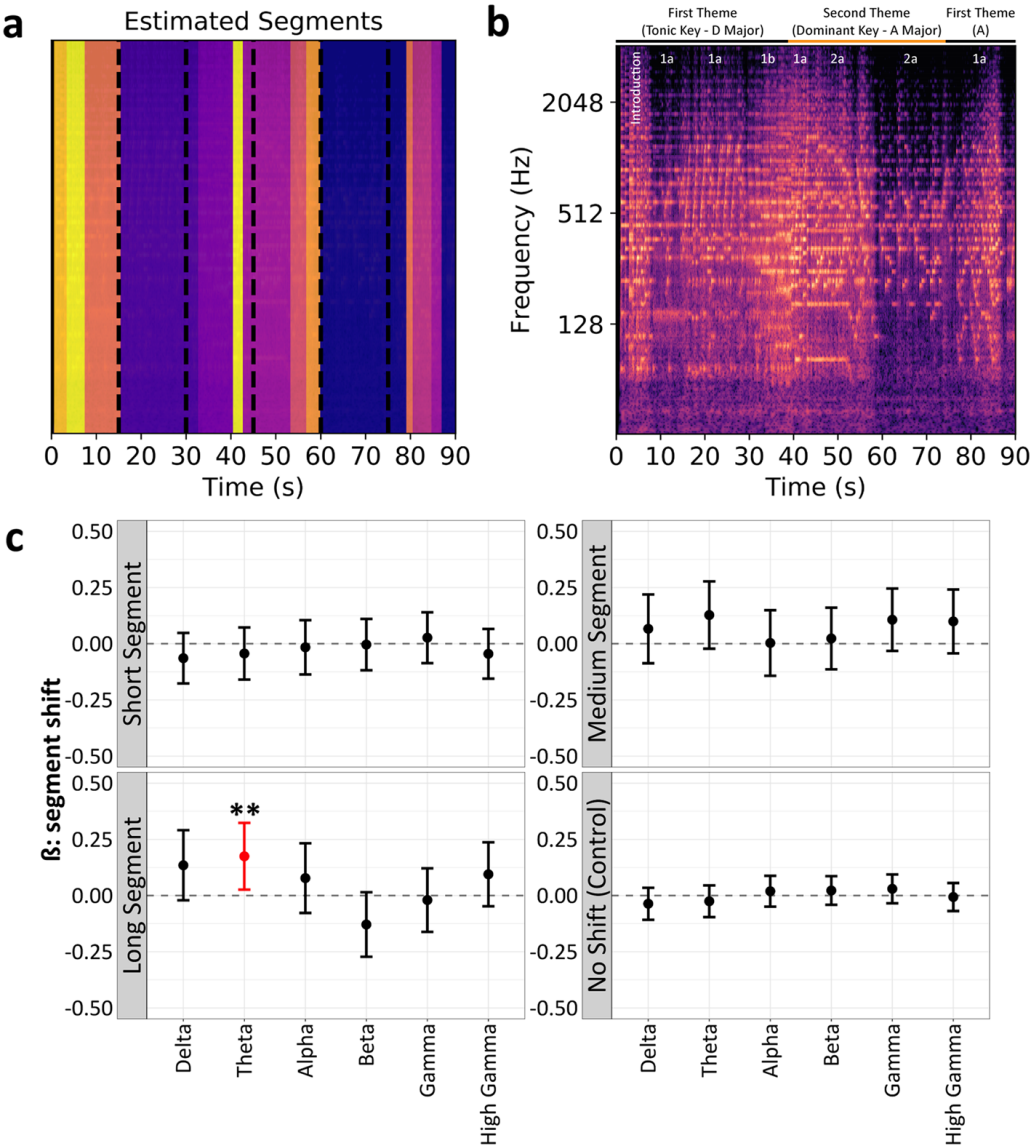
Enhanced frontal theta activity during shifts from long musical segment boundaries. (**a**) Detected segments for Mozart’s K448 with dashed lines indicating 15-s window boundaries used for our IED-related analyses. (**b**) Constant-Q spectrogram of K448 overlay with annotations from a theoretic analysis of the exposition of the first movement of Mozart’s K448 (i.e., K448’s musical score). (**c**) An assessment of the association between different Stereo-EEG powerbands and musical segment shifts (short = 3-s or less, medium = 3–10 s, long = 10-s or more, control = periods of no segment shift). There was a significant association between increased frontal theta activity and transitions from longer musical boundaries (ß = 0.17, % increase = 19.10, *p* = 0.002). All other powerbands and musical segment categories showed nonsignificant associations. ß values above zero reflect increased power, and ß values below zero reflect decreased power during a musical segment shift. Significance at *p < 0.05, **p < 0.01, ***p < 0.001.

An acoustic analysis of the nested structural components of Mozart’s K448 revealed several segment boundaries for repeated sequences (Fig. 5a). These segment boundaries coincided with a professional musician’s annotations of the musical score (Fig. 5b). We investigated the association between segment boundaries and frontal activity, as this was the only region with significant IED effects. There was a significant association between increased frontal theta activity and transitions from longer musical boundaries (ß = 0.17, % increase = 19.10, *p* = 0.002) (Fig. 5c). All other powerbands and musical segment categories showed nonsignificant associations (Fig. 5c). Repeating this procedure with the filtered version of K448 revealed a nonsignificant relationship between all powerbands and musical segment categories in the frontal cortex (Supplementary Fig. S1a); this suggests that the broad structural components of K448 are preserved but altering the frequency structure of the original composition attenuated neural responses. Applying this procedure to Wagner’s composition also revealed nonsignificant associations between all powerbands and musical segment categories in the frontal cortex (Supplementary Fig. S1b).

## Discussion

In this study, we observed an association between noninvasive musical stimulation and reductions in intracranial interictal activity in persons with refractory epilepsy. Our study is one of two extant in the literature that examined the “Mozart K448 effect” in an adult population with intracranial recordings^33^. We advance past findings by testing if there was a minimum length of exposure needed to elicit this therapeutic effect and if novel music alteration methods could enhance this phenomenon. To our knowledge, this is the first study to systematically evaluate the relationship between musical segment boundaries and spectral power changes as they relate to the “Mozart K448 effect” in persons with epilepsy.

Although previous studies have investigated the role of K448 on interictal and ictal activity^9,11,12,15,16,18,19,34^, our study further demonstrates this effect using intracranial Stereo-EEG implants in an adult population with refractory epilepsy. We showed that the original version of K448 could effectively reduce IED rates with exposures as short as 30-s. The 66.5% average global IED reduction observed in our study is consistent with the upper limit of IED responses to K448 reported in the past. For instance, Lin et al.’s most recent work showed a 79.4 ± 20.0% average reduction in IEDs detected with scalp-EEG after one month of K448 exposure^9^. A meta-analysis of other scalp-EEG studies also reported an average IED reduction of approximately 35% during Mozart’s K448^13^. Recently, Štillová et al. (2021) used intracranial recordings to report a median IED reduction of 32%^33^. The enhanced effect observed in our study could be explained by our use of Stereo-EEG instead of scalp-EEG^9–12,18^, as Stereo-EEG is more reliable for detecting “true” intracranial interictal activity^20,21^, or by differences attributable to heterogeneous sample populations immanent with intracranial studies (i.e., high variability between subjects with refractory epilepsy).

Our observation of no carry-over of the “Mozart K448 effect”, evident by no effect persisting into the post-stimulus periods, showed that 90-s of exposure was likely too short for a lasting impact on neural activity. This contrasts with previous work, which reported significant effects on seizure frequency in the post-treatment follow-up^34,35^. For instance, Bodner et al.^34^ showed a significant 33% reduction in seizures that persisted into the follow-up phase after K448 exposure, indicating that brief exposures of 90-s or less may invoke a less pronounced neural response than those witnessed with longer stimulus durations^9–11,19^. Nonsignificant observations for Group 15 also suggest a weaker neural response with transient exposures. Nonetheless, our null IED findings for the altered music, music matched to K448, and music from subject-preferred genres reinforce the claim that there might be something special about Mozart’s original composition, especially for interacting with the pathology of epilepsy. These findings reveal the importance of stimulus duration and encourage future work to determine the optimal duration of music for generating enduring therapeutic responses.

Models investigating less pathologic brain regions (Non-SOZ) highlighted the bilateral frontal cortices as regions important for the “Mozart K448 effect”. This agrees with past observations that listening to music was associated with increased activation of prefrontal cortices^36–39^. Such as Mansouri et al.’s (2017)^39^ observation that high-tempo music activated prefrontal cortical areas, while transcranial direct current stimulation (tDCS) over prefrontal regions negated the influence that music had on executive functions. These current findings also agree with Rauscher et al.’s (1993)^14^ original observation that K448 enhanced spatial–temporal working memory, a process directly linked with dorsal frontal activation.

We applied a structural decomposition technique to the original version of Mozart’s K448 to identify local and long-range nested structures based on the composition’s harmonic and timbral features. Our investigation revealed enhanced frontal theta power following shifts from longer musical segments (i.e., 10-s or more) that was not present during transitions from shorter musical segment boundaries and during all transitions within the filtered version of K448 and Wagner’s *Lohengrin Prelude to Act* I. These findings are concordant with past music research, which demonstrated that pleasant music was associated with increased frontal theta power^40–43^. Previous studies even proposed that frontal theta may represent a gating mechanism for the passage of information to the limbic system^41^ and showed that the limbic system’s activity directly correlates with theta oscillations in the frontal cortex, particularly in response to emotionally arousing musical stimuli^40,42,43^.

Further evidence for the relationship between music and frontal emotion networks is provided by Tillmann et al.’s (2003)^44^ fMRI study, which showed enhanced activation of the bilateral inferior frontal regions for unexpected targets. More specifically, the structural syntactic relations between musical events led to increased bilateral frontal activation, where greater activation was correlated with processing incoherent, unexpected events^44^. In 2017, Arjmand et al.^45^ augmented these findings by showing that unexpected changes in musical features, such as intensity and tempo, activated frontal brain regions linked with positive emotional responses. In conjunction with our current findings, this suggests that the generation of neural predictions about musical features may depend on both the duration of exposure and transitions from prolonged segments within the musical stimulus—as this may be driving enhanced activation of internal emotion networks regulated by frontal cortices.

Our theory for the “Mozart K448 effect” raises a critical distinction between subjective emotional responses to music and internal, evoked emotional brain responses. This is supported by our findings of nonsignificant IED changes for musical pieces from the subject preferred genre. Additional support for this theory is provided by Hughes et al.’s (1998) landmark study^15^, which showed that K448 reduced IED activity even in subjects in a comatose state. In this study, Hughes et al. (1998) also showed that theta activity decreased in the central areas, while delta activity increased in frontal areas during K448^15^. Our increase in frontal theta is comparable to their increase in frontal delta, whereby the slight difference in frequency may be associated with their use of scalp-EEG, which is inferior at detecting higher frequency components. Era-related differences in EEG hardware, study protocols, subject populations, and analytical control (i.e., our control for the influence of the SOZ and ASM status) could further explain these discrepancies.

In revealing that the musical structure of K448 may be contributing to its therapeutic effect, we shed light on a new theory for the “Mozart K448 effect” in epilepsy: the musical structure defined by the sonata form may elicit positive emotional responses that may be important for anti-epileptic effects. This is further supported by past observations that the only other composition with anti-epileptic properties was Mozart’s Piano Sonata in C Major (K545)^18^. We also confirmed the importance of other musical features, such as the stimulus’s frequency components, by showing that the filtered version of K448 failed to elicit a therapeutic response^10,33^. Thus, despite similar broad structural components, the filtered version of K448 may have decreased emotional salience (i.e., frequency distortions made it less acoustically pleasurable), resulting in a reduced likelihood of developing internal musical predictions and engaging emotionally with the piece.

A theoretical evaluation of the first 90 s of Mozart’s K448 shows that it is structurally organized by contrasting melodic themes, each with its own underlying harmony. This is contrasted by the first 90 s of Wagner's *Prelude to Act I of Lohengrin*, which has no recognizable melodies. Called “the first piece of hypnosis by music”^46^, and one of Wagner's most popular musical works, the selection consists solely of static chords that are held for long durations before small shifts in instrumentation and harmony occur. Thus, the structure of Wagner’s selection is organized by subtle and gradual changes instead of contrasting melodic themes, as seen in Mozart’s K448. This work was selected to control for the effect of melody-with-harmony versus harmony alone. It also underscores the importance of selecting proper negative musical controls to systematically uncover components essential for the “Mozart K448 effect” and enhance the validity of experimental findings. Future work will focus on using additional musical controls to further identify components of K448 that are essential for its therapeutic effect. That is, we will focus on analyzing carefully curated musical controls that are specifically matched to certain features of K448 (e.g., frequency, musical structure, musical segment durations) to isolate components essential for beneficial responses. This may enable us to replicate the “Mozart K448 effect” with other musical stimuli through (1) algorithmically searching for stimuli with matching essential components or (2) adding essential components with secondary signal alterations.

Several factors limit the implications of this current study. Our automated IED detection could introduce bias; however, it provided a means for objectively marking IEDs in light of the discordance between human reviewers^2^. We are also missing surgical outcome data and ASM blood levels, which may further bias IED-related findings. The relatively small number of Stereo-EEG subjects presents another limitation, which could be responsible for some of the non-significant results presented. However, our sample size was typical for most intracranial studies, which require fewer subjects due to significantly larger effects detectable with intracranial recordings. Our study also provides the foundation and methodology for future multicenter studies that can recruit a larger number of subjects with refractory epilepsy. We did not run the same experiment in all subjects, as we did not consider the importance of stimulus duration in our initial study, which would have been ideal. Thus, Group 15 offers complementary evidence for Group 90’s findings in different persons with refractory epilepsy. Another general limitation is that our study did not specifically measure if positive, subjective emotions were evoked while subjects listened to Mozart’s K448. Nonetheless, we provide evidence for internal representations of emotions through previously reported neural patterns. That is, our findings were concordant with the literature in showing frontal activation following shifts in musical expectations. We also demonstrated that while persons with epilepsy do not generally listen to classical music, it does not preclude them from enjoying and benefitting from Mozart’s K448 at an internal, neural level.

In conclusion, the current findings demonstrate that musical stimulation with Mozart’s K448 may reduce IED rates inside and outside the seizure onset zone in persons with refractory epilepsy. We show that the “Mozart K448 effect” has a lower limit of approximately 30-s for evoking therapeutic neural responses and provide evidence for the preferential reduction of IEDs in bilateral frontal regions, with implications for the activation of emotion networks regulated by the frontal cortex. Our data suggest a strategy for the noninvasive modulation of intracranial interictal activity, which may alleviate IED-related comorbidities. They support the future investigation of other sonatas with similar structural characteristics to Mozart’s K448, as they may hold therapeutic potential for epilepsy. Ultimately, our study provides insight into intracranial mechanisms that may be important for the anti-epileptic properties of Mozart’s K448.

## Material and methods

### Participants

Sixteen subjects undergoing intracranial electroencephalographic monitoring for the clinical treatment of refractory focal epilepsy participated in this study (Table 1). All subjects reported little to no previous musical training and limited exposure to classical music. The research protocol for this study was approved by the Committee for the Protection of Human Subjects (CPHS#: 12495) at Dartmouth College, and informed consent was obtained from each subject. All methods were carried out in accordance with the relevant guidelines and regulations of this ethics committee. Electrophysiological data were collected from depth electrodes implanted within the brain parenchyma to best localize epileptogenic regions.

### Stereo-EEG data

Stereo-EEG macroelectrodes recorded electrophysiological data at sampling rates ranging from 500 to 1500 Hz (Natus Medical Inc.). Recording channels were excluded if the raw signal was greater than two standard deviations from the median value across channels to remove non-physiological artifacts or if channels were outside of co-registered brain regions. Stereo-EEG data were band-pass filtered from 1 to 50 Hz, re-referenced to an average referential montage, excluding the channels with artifacts, then resampled at 256 Hz.

### Anatomical localization

For all subjects, pre-implant T1-weighted and T2-weighted MRI images were co-registered with postoperative computed tomography (CT) to obtain the position of small-spacing Stereo-EEG depth electrodes. Freesurfer and the Desikan–Killany atlas were used for hippocampal subfield localization and cortical parcellation, and then final electrode positions were manually reviewed by two neuroradiologists^47–50^. Electrodes were finally reclassified into the following broader regions: left superior temporal cortex (STC), right STC, left middle temporal cortex (MTC), right MTC, left mesial temporal cortex (Mesial), right Mesial, left frontal cortex (FC), and right FC.

### Automated spike detection

An automated template-matching detector was used for the detection of all IEDs in this study. This detector was previously validated and performed comparably to clinicians at Dartmouth–Hitchcock (DH) and other published detectors^4,51,52^. The detector used the following pipeline to mark IEDs: (1) cross-correlate a 60-ms triangular template with preprocessed Stereo-EEG, (2) normalize the cross-correlation by the median standard deviation from 1-s sliding windows, (3) calculate the absolute value of the normalized cross-correlation, and (4) mark local peaks above a specified detection threshold as IEDs (Fig. 1). We then collapsed temporally overlapping detections into a single marked event and excluded IEDs occurring within 2-s of another IED to account for bursts of spikes. An illustration of this pipeline and sample IED detections are provided in Fig. 1.

### Spectral power

Due to spectral perturbations associated with IEDs, we first divided task epochs into 1-s segments, then rejected all task epochs within 3-s of an IED. Our goal was to assess spectral power between brief pre- and post-segment boundaries (i.e., musical transitions), so we used the multitaper spectral analysis method, which convolved orthogonal Slepian sequences with the Stereo-EEG signal to provide new periodograms. A final spectrum, obtained by averaging over all the periodograms, was used to calculate the average spectral power within the following canonical frequency bands: delta (2–4 Hz), theta (4–7 Hz), alpha (8–12 Hz), beta (12–30 Hz), gamma (25–40 Hz), and high gamma (40–100 Hz)^53^. We log-transformed and z-scored the power within each experiment session for each electrode for each subject, then averaged power values into non-overlapping 1-s time bins for each trial^54^.

### Musical boundary detection

We defined musical boundaries using a technique developed and detailed by McFee and Ellis (2014)^49^. Specifically, we applied techniques that operate on the graph Laplacian to identify repeated patterns in the musical composition to create block structures in the spectrum based on expanded diagonal bands of a self-similarity matrix^49^. This music information retrieval technique identified a hierarchical structure using (1) harmonic features for long-range repetitions and (2) timbral features for short-range patterns to define structural boundaries in the music^55,56^. Spectral clustering was performed using k-means clustering (k = 10) with the normalized eigenvectors of the symmetric normalized Laplacian. The input signal was sampled at 22,050-Hz (mono) and analyzed with a 2048-sample FFT window and 512-sample hop. Music segments were reclassified into the following categories: short (3 s or less), medium (3–10 s), and long (10 s or more). These musical boundaries demarcated shifts in musical themes within the 90-s clips, allowing us to test the hypothesis that frontal activity (i.e., emotion networks) was associated with positive reward prediction errors, observable during transitions out of longer musical segments^28^. That is, we hypothesized that (1) longer segments with a similar musical structure were required for subjects to develop expectations, then (2) violations of those internally generated expectations would be correlated with the preferential activation of emotion networks.

### Auditory task

The first group of subjects (referred to as “Group 15”) was presented with blocks of 15-s clips of distinct auditory stimuli, presented in a random order for each subject session through sampling without replacement (Fig. 2a). Auditory stimuli were followed by a 15-s control period that consisted of normal ambient room noise (i.e., silence in the acoustic speaker). Although four different auditory stimuli were presented to this group, we only examined Mozart’s Sonata for Two Pianos in D Major (K448) and Mozart’s Sonata for Two Pianos in D Major that was amplitude modulated with a 40 Hz sinusoid (modulatedK448), using the control period as a reference.

The second group of subjects (referred to as “Group 90”) was presented with blocks of 120-s clips of musical stimuli, presented in a random order for each subject session through sampling without replacement (Fig. 2b). During the last 30-s of each auditory stimulus, each subject was presented with a simultaneous attention task. Again, the control period following each auditory stimulus consisted of normal ambient room noise, followed by a Boolean question assessing whether the subject attended to the auditory stimulus. Nine different musical stimuli were presented to this group. However, our primary analysis focused on Mozart’s Sonata for Two Pianos in D Major (K448) and Mozart’s Sonata for Two Pianos in D Major that was band-pass filtered to boost gamma frequencies (filteredK448), using the control period as a reference. We also included an orchestral version of Wagner’s *Lohengrin Prelude to Act I* and violet noise as two control stimuli. We focused on Wagner’s *Lohengrin Prelude to Act I* because it had similar general popularity to Mozart’s K448 within the classical genre and included a comparable number of musical boundaries identified by our segmentation method with significantly fewer long musical segments (SFig. 1). It also paralleled Lin et al.’s (2010) use of a string version from the classical genre^10^, making it an ideal negative control. Violet noise was selected as a non-musical auditory control because it has a power density that increases per octave, weighting it towards the top of the spectrum. This makes it an ideal negative control for testing our hypothesis that lower frequencies (e.g., gamma boosted) are important for reducing IED rates.

Each experiment session had a typical duration of 25-min and was repeated twice per subject on average. The first 15- and 90-s from Mozart’s K448 (*Allegro con spirito*) were used for this study. We utilized a Roland C30 loudspeaker to deliver the auditory stimuli at a comfortable sound level determined by the subject, ranging from 60 to 70 dB. All experiment sessions were performed at least four hours after the most recent seizure and at least 24 h post-implantation.

### Auditory stimuli

A complete list of the auditory stimuli presented to Group 15 may be found in our previous publication^22^. Apart from the original version of K448, altered version of K448, Wagner, and violet noise, other auditory stimuli presented to Group 90 were Frederic Chopin's Bolero in C—Op. 19 for piano, performed by Nikita Magaloff (“Classical T”); Franz Liszt’s Piano Sonata in B Minor, 1st movement: Lento assai—Allegro energico, performed by Leslie Howard (“Classical N”); and three songs from a preferred musical genre. That is, each subject was asked to select a preferred genre after sampling a preselected list of songs from the classical country (Tumbling Tumble Weeds by Sons of the Pioneers [“Preferred T”]; Barbara Allen by Bradley Kincaid [“Preferred N”]), heavy metal (Jugulator by Judas Priest [“Preferred T”]; Just For by Nickelback [“Preferred N”]), and rock and roll (Na Na Hey Hey Kiss Him Goodbye by Steam [“Preferred T”]; Peggy Sue by Buddy Holly [“Preferred N”]) genres. Within this subject-preferred genre, we also altered the “Preferred N” song by boosting lower frequencies (“Preferred N Altered”). The “T” or “N” following each song label indicates whether the gamma-range auditory modulation spectrum of that song matched (“T”) or did not match (“N”) that of K448 using the amplitude-modulation-analysis toolbox [https://github.com/MuSAELab/amplitude-modulation-analysis-matlab]. The modulation-spectrum analysis was run on a large musical corpus to identify maximally matching (“T”) and divergent (“N”) musical pieces to be investigated in our study. All selected songs, both matching and divergent, were additionally tempo-matched to the mean tempo of their respective genre using the librosa library to implement automatic tempo extraction^57^. “Altered” indicates signals with secondary gamma modulations. The first 90 s from each musical selection were used in our study. The order of stimuli presented for each subject session is provided in SFig. 2.

### Statistical analysis

We used Generalized Estimating Equations (GEE) log-linear regression models with extra-Poisson variance assumptions to determine if there was a difference in the normalized IED rates across all control periods between Group 15 and Group 90 and to compare pre-experiment baseline IED rates with control IED rates. In these models, the within-subject association was specified in terms of an unstructured pairwise correlation pattern, and the variances of the counts were adjusted with sandwich estimators. To ensure that natural transient fluctuations in IEDs did not drive subsequent findings, we z-scored IED rates within each subject, then examined if there was a difference between any of the experiment trials using a repeated measures analysis of variance (RM-ANOVA). An a priori power analysis used the effect sizes reported by Lin et al.’s (2014) most recent work^9^, an alpha of 0.05, and power of 0.90 to determine that a minimum of eight subjects were required to evaluate the main objective of this study.

Similar GEE log-linear regression models, retaining the same previous assumptions about the variance and correlation, were used to determine if there was a significant global normalized IED rate reduction during the original K448 relative to the average control period both inside (SOZ) and outside (Non-SOZ) of the seizure onset zone. Analogous models were used to assess the altered versions of K448 relative to the control period. We subsequently partitioned Group 90’s data from the original K448 into six 15-s windows, then evaluated each window independently. Due to the reduced number of IEDs per 15-s time window, we used Generalized Linear Mixed Models (GLMM) that assumed a zero-inflated Poisson distribution with random slopes and intercepts for each subject and an offset term for exposure time. We repeated this procedure for Group 90’s data during the control stimuli, using similar models to evaluate each window independently. A post hoc paired-sample Wilcoxon test was used for pairwise comparisons to determine if there was a significant difference between specific windows of interest. Another post hoc analysis used analogous GEE log-linear regression models to assess all other musical stimuli presented to Group 90.

We evaluated subject-specific changes, between control and K448 conditions, in the rate of IEDs for specified brain regions outside of the SOZ. We fit the same mixed effects log-linear regression model separately for each region using region-specific IEDs with the following predictors: ASM status and session time, including random slopes and intercepts for the within-subject factor, and an offset term for exposure time. This model assumed IED rates had a zero-inflated Poisson distribution and controlled for natural heterogeneity between subject IED rates and expected changes in IED rates over time.

The presence of prolonged segment boundaries (i.e., transitions out of persistent structural patterns) in the time windows corresponding to significant IED reductions inspired our subsequent analyses, which focused on determining if powerband shifts in the frontal cortices were associated with musical segment boundaries. These analyses were also motivated by (1) our finding that bilateral frontal regions were preferential for the “Mozart K448 effect” and (2) the theory of music and emotion, whereby positive emotional responses are thought to be correlated with the neural processing of acoustic patterns in frontal brain regions^44^. GLMM were used to assess the statistical relationship between shifts in musical boundaries and power in the frontal cortex. These models compared the average frontal power from 1-s before a segment boundary to the average frontal power from 1-s after a segment boundary. We repeated this analysis for frontal brain regions during exposure to the filtered version of K448 and Wagner’s composition as a validation.

Of note, past studies relied primarily on paired t-tests^9–11^ or its nonparametric alternative, the paired samples Wilcoxon test^12,33^ to assess changes in interictal activity. For our group-level analyses, we used a semiparametric test, the GEE, which is similar to conventional paired tests but is generally more robust, as it is less affected by departures from parametric assumptions^58^. Differences in statistical power are especially notable when comparing the GEE with the Wilcoxon test, as the latter relies on ranks rather than actual count outcomes. In the setting of our experiment, the GEE and paired t-test are essentially equivalent, except that the GEE uses the asymptotic normal distribution for inference rather than the t distribution, making it the preferred method for a smaller sample size^58^.

Relative Risks (RRs) were computed from odds ratios (ORs) for better estimations and were reported with 99.9% confidence intervals to reflect the significance of corrected *p* values^59^. All models included offset terms to account for different task window durations while also controlling for the influence of anti-seizure medication (ASM) status, unique session time, and subject heterogeneity. The family-wise error rate (FWER) was controlled at 0.05 using Bonferonni correction unless otherwise specified.

## Supplementary Information

Supplementary Figure 1.

Supplementary Figure 2.

## Data Availability

Deidentified Stereo-EEG data are available upon reasonable request.
