# Supplementary material for: Musical components important for the Mozart K448 effect in epilepsy
Source: Sci Rep. 2021 Sep 16;11:16490. doi: 10.1038/s41598-021-95922-7 (PMC8446029; doi:10.1038/s41598-021-95922-7)
Supplement: Supplementary file 1 — Supplementary Figure 1. [file 41598_2021_95922_MOESM1_ESM.pdf]

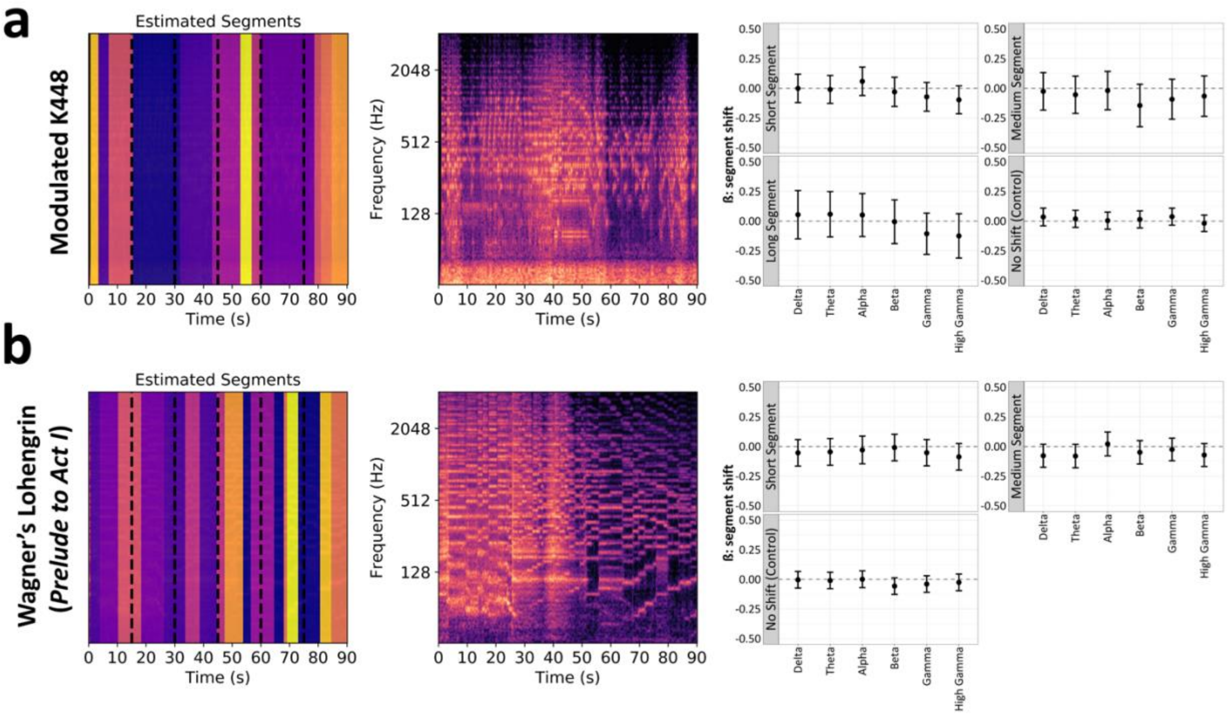

Supplementary Figure 1. Evaluation of spectral power and segment shifts in other compositions. (a) The filtered version of K448 (gamma frequency boosted) revealed nonsignificant associations between all spectral powerbands and musical segment categories in the frontal cortex. (b) Wagner's Lohengrin (*Prelude to Act I*) also revealed nonsignificant associations between all powerbands and musical segment categories in the frontal cortex. Significance at *p < 0.05, **p < 0.01, ***p < 0.001.
