# Supplementary material for: Musical components important for the Mozart K448 effect in epilepsy
Source: Sci Rep. 2021 Sep 16;11:16490. doi: 10.1038/s41598-021-95922-7 (PMC8446029; doi:10.1038/s41598-021-95922-7)
Supplement: Supplementary file 2 — Supplementary Figure 2. [file 41598_2021_95922_MOESM2_ESM.pdf]

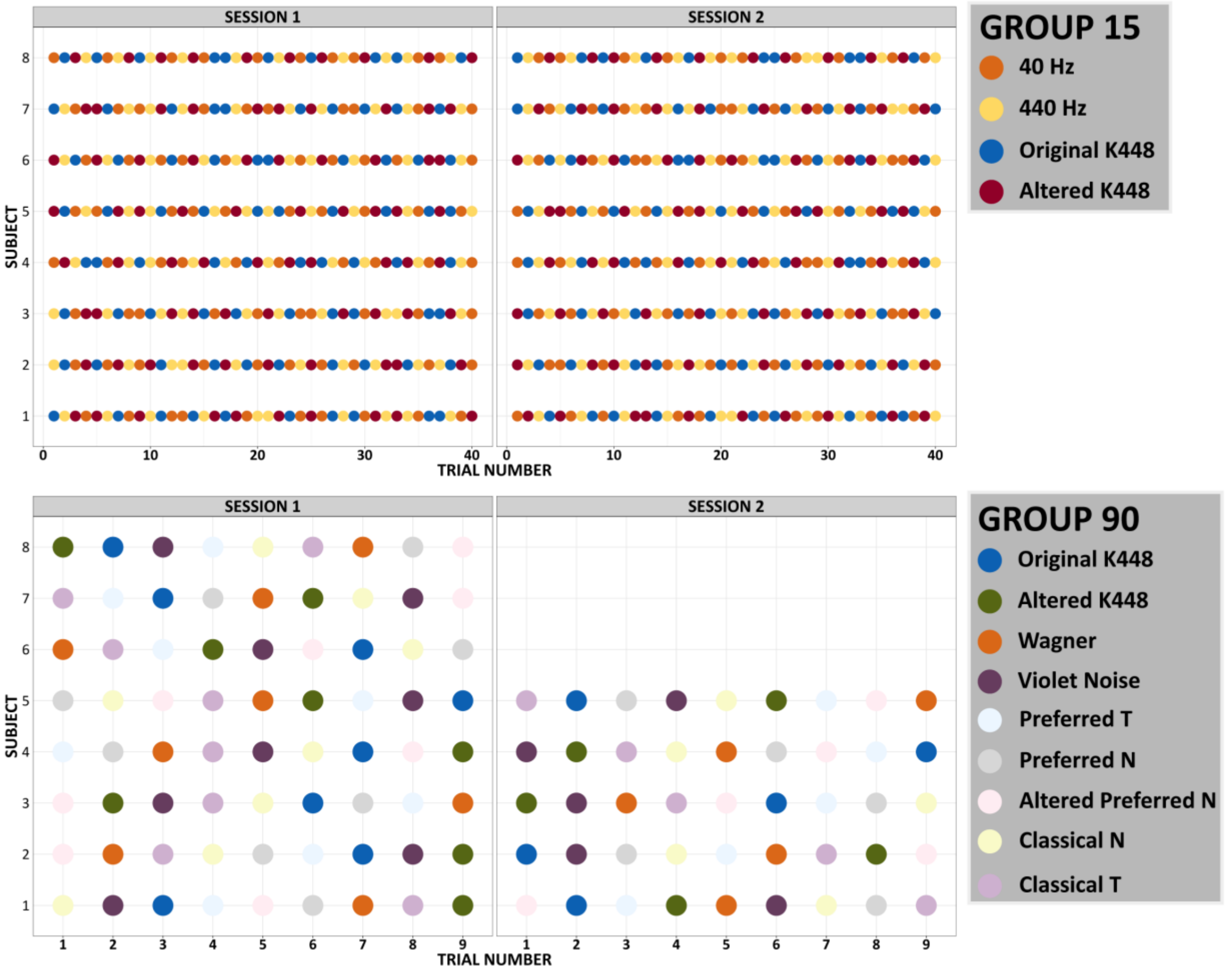

Supplementary Figure 2. Order of presented stimuli. Auditory stimuli were randomly sampled without replacement from a preselected corpus. The colored points correspond to different auditory stimuli and depict the order of stimuli presented for each unique subject session. The "T" or "N" following each song label indicates whether the gamma-range auditory modulation spectrum of that song matched ("T") or did not match ("N") that of K448. All selected songs were tempo-matched to the mean tempo of the respective genre.
